# Author Correction: Inhibition of acetylation of histones 3 and 4 attenuates aortic valve calcification

**DOI:** 10.1038/s12276-024-01216-z

**Published:** 2024-05-15

**Authors:** Jia Gu, Yan Lu, Menqing Deng, Ming Qiu, Yunfan Tian, Yue Ji, Pengyu Zong, Yongfeng Shao, Rui Zheng, Bin Zhou, Xiangqing Kong, Wei Sun

**Affiliations:** 1 Department of Cardiology, The First Affiliated Hospital of Nanjing Medical University, 300 Guangzhou Road, 210029 Nanjing, PR China; 2 Department of Cardiothoracic Surgery, The First Affiliated Hospital of Nanjing Medical University, 300 Guangzhou Road, 210029 Nanjing, PR China; 3 Departments of Genetics, Pediatrics, and Medicine (Cardiology), The Wilf Cardiovascular Research Institute, The Institute for Aging Research, Albert Einstein College of Medicine, Bronx, NY 10461 USA

Correction to: *Experimental & Molecular Medicine* (2019) 51(7):1-1410.1038/s12276-019-0272-9, published online 10 July 2019

In this article, the order that the authors appeared in the author list was incorrect.

After online publication of this article, the authors noticed an error in the images of Fig. 1B section. The correct figure should have appeared as shown below.
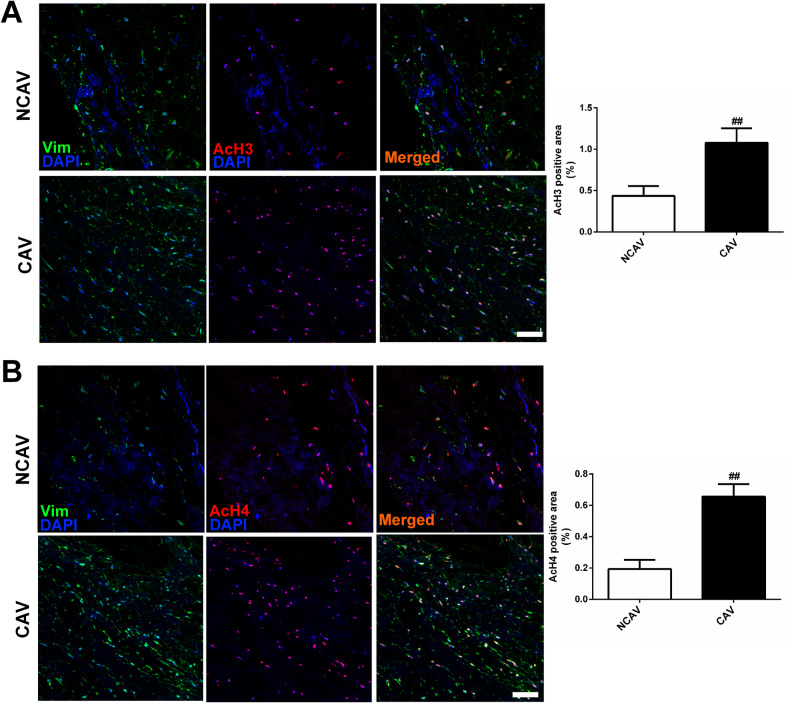


The authors apologize for any inconvenience caused.

The original article has been corrected.

